# Forecasting Japan's Physician Shortage in 2035 as the First Full-Fledged Aged Society

**DOI:** 10.1371/journal.pone.0050410

**Published:** 2012-11-30

**Authors:** Koichiro Yuji, Seiya Imoto, Rui Yamaguchi, Tomoko Matsumura, Naoko Murashige, Yuko Kodama, Satoru Minayo, Kohzoh Imai, Masahiro Kami

**Affiliations:** 1 Department of Internal Medicine, Research Hospital, The Institute of Medical Science, The University of Tokyo, Minato-ku, Tokyo, Japan; 2 Laboratory of DNA Information Analysis and Laboratory of Sequence Analysis, Human Genome Center, The Institute of Medical Science, The University of Tokyo, Minato-ku, Tokyo, Japan; 3 Division of Social Communication System for Advanced Clinical Research, The Institute of Medical Science, The University of Tokyo, Minato-ku, Tokyo, Japan; Tokai University, Japan

## Abstract

**Introduction:**

Japan is rapidly becoming a full-fledged aged society, and physician shortage is a significant concern. The Japanese government has increased the number of medical school enrollments since 2008, but some researchers warn that this increase could lead to physician surplus in the future. It is unknown how many physicians will be required to accommodate future healthcare needs.

**Materials and Methods:**

We simulated changes in age/sex composition of the population, fatalities (the number of fatalities for the consecutive five years), and number of physicians from 2010 to 2035. Two indicators were defined: fatalities per physician and fatalities by physician working hour, based on the data of the working hours of physicians for each tuple of sex and age groups. We estimated the necessary number of physicians in 2035 and the number of new physicians to maintain the indicator levels in 2010.

**Results:**

The number of physicians per 1,000 population is predicted to rise from 2·00 in 2010 to 3·14 in 2035. The number of physicians aged 60 years or older is expected to increase from 55,375 (20% of physicians) to 141,711 (36%). In 2010 and 2035, fatalities per physician were 23·1 and 24·0 for the total population, and 13·9 and 19·2 for 75 years or older, respectively. Fatalities per physician working hour are predicted to rise from 0·128 to 0·138. If working hours are limited to 48 hours per week in 2035, the number of fatalities per physician working hour is expected to be 0·196, and the number of new physicians must be increased by 53% over the current pace.

**Discussion:**

The number of physicians per population continues to rise, but the estimated supply will not fulfill the demand for healthcare in the aging society. Strategies to increase the number of physicians and improve working conditions are urgently needed.

## Introduction

The population is aging rapidly in high-income countries. For example, Japan's total fertility rate was 1·37 in 2009, while the average life expectancy was 77·1 years for males and 84·4 years for females. People aged 65 or older accounted for 23·1% of the total population in Japan in 2010 [1]. This figure will increase to 38·7% in 2035 and represents the greatest proportion of elderly among high-income countries [1].

The number of Japanese physicians is low (2·15 per 1,000 population) compared with other high-income countries in the Organisation for Economic Co-operation and Development (OECD; mean  = 3·00) [2]. Because population aging drives healthcare demand, [3] the physician shortage in Japan will likely become a bigger problem in the future.

The Japanese government strictly restricts physician supply, and Japanese medical schools impose a maxim level on medical school enrollment. As a result, a shortage of physicians, especially obstetricians and gynecologists, has emerged as a serious social issue [4]–[8]. Physicians' refusing to see high-risk patients and “bouncing” patients to other hospitals have attracted public concern [9]. Medical school enrollment has risen 16% (1,221 students) from 2008 to 2011 by the Japanese Government. The Japanese Government predicts that the number of physicians per 1,000 population will rise in the future. However, the Japan Medical Association and some researchers warn that the increase in physician numbers could lead to a surplus of physicians in the future and physician maldistribution should be solved [10]–[13].

**Figure 1:**
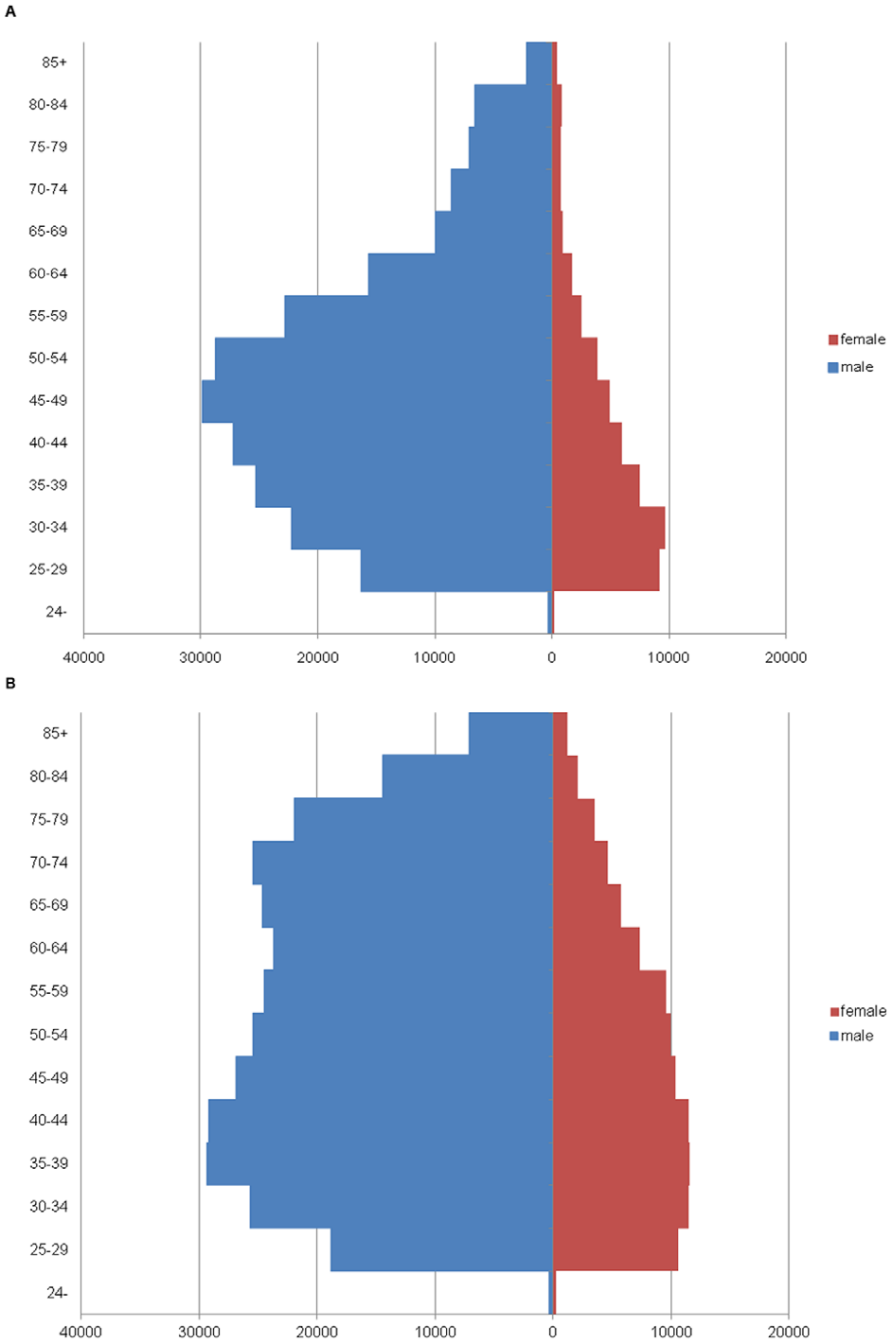
Physicians' population pyramids. Physicians' population pyramids for 2010 (panel A) and 2035 (panel B).

However, these predictions are associated with several problems. First, overwork of physicians has not been considered. Japanese physicians work an average of 70·6 hours per week (85 hours for those in their late 20 s and 48 hours for those in their 60 s) [14]. Japanese physicians have been reported to experience exhaustion, sudden death, and suicide from overwork [15]. It is important to restrict working time from the point of view of patient safety and physicians' health [16]. In Europe, working hours of junior doctors are limited to 48 hours per week by the EU Working Time Directive from 2009 [17]. In the United States, the Accreditation Council for Graduate Medical Education implemented duty hour limitations in 2003, contributing to improvement of patient safety, resident safety, and education [18].

**Table 1:** Changes in simulated paramaters, 2010 and 2035.

	2010	2035	increase rate
**The number of physicians per 1,000 population**	2·00	3·14	57%
**The number of populations**	127,176,445	110,679,406	−13%
**The total number of physicians**	271,897	397,290	46%
The number of physicians aged 75 years or younger	254,126	347,103	37%
The number of physicians aged 75 years or older	17,771	50,187	182%
The number of physicians aged 60 years or younger	216,522	255,579	18%
The number of physicians aged 60 years or older	55,375	141,711	155%
The number of male physicians	222,784	297,483	34%
The number of female physicians	49,113	99,807	103%
**practicing physicians' working hours per 1,000 population**			
no limitation/current working hours	139·2	209·8	51%
limited to 60 hours/week		178·2	28%
limited to 48 hours/week		147·7	6%
**The total number of fatalities for consecutive five years**	5,881,151	8,336,263	42%
The number of fatalities for 75 years or older	3,529,540	6,650,448	88%
The number of fatalities for 74 years or younger	2,351,611	1,685,815	−28%
**The number of fatalities per practicing physician**	23·1	24·0	4%
fatalities per practicing physician for 75 years or older	13·9	19·2	38%
fatalities per practicing physician for 74 years or younger	9·2	4·8	−48%
**The number of fatalities per practicing physician working hour**			
no limitation/current working hours	0·128	0·138	8%
limited to 60 hours/week		0·162	27%
limited to 48 hours/week		0·196	53%

Second, changes in sex and age composition have not been considered when discussing physicians' working hours. The number of practicing female physicians was 10,218 (9·3%) and 51,997 (18·1%) in 1965 and 2008, respectively [19]. Female doctors work fewer hours than their male counterparts (78 vs. 85 hours in their late 20 s and 40 vs. 48 hours in their 60 s) [14]. The supply of and demand for Japanese physicians have not been projected based on the actual physicians' workforce and patient/physician age structure.

**Figure 2:**
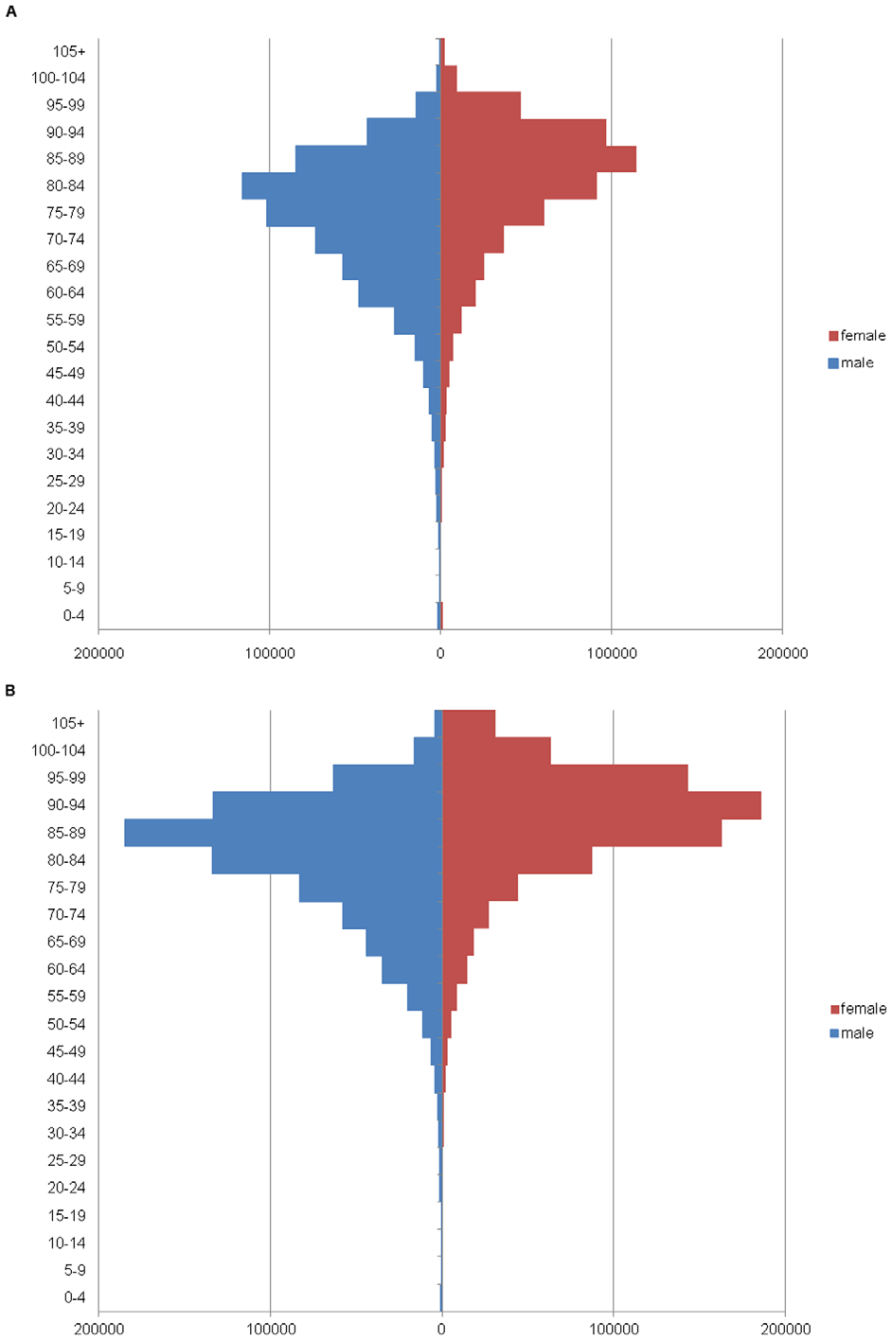
Fatality pyramids. Fatality pyramids for 2010 (panel A) and 2035 (panel B).

An aging population is a common problem in high-income countries, and Japan will likely become a model of future healthcare systems. To forecast the balance between physician supply and healthcare needs, we simulated population, fatalities, and number of physicians in Japan from 2010 to 2035.

## Materials and Methods

We simulated changes in population between 2010 and 2035 based on data provided by the National Institute of Population and Social Security [1]. The simulations were performed every 5 years from 2010 to 2035 for all of Japan and for each of the 47 prefectures. The number of fatalities for the consecutive five years (An estimate of the number of deaths in the next five years) was calculated based on the future life chart [1]. Let *x_isj_*(*t*) be the population of the *i*th prefecture (*i* = 1,...,47), sex *s* (*s* = 1 for male and *s* = 2 for female), and *j*th the age group at year *t*. The population at year *t*+5 is predicted by
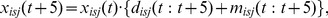
(1)where *d_isj_*(*t* : *t*+5) and *m_isj_*(*t* : *t*+5) are the rates of survival and migration during the 5 years from year *t* to *t*+5, respectively. We applied this concept to predict the distribution of the number of physicians at year *t*+5 from that at year *t*, denoted by *y_isj_*(*t*).

Simulation of the number of physicians by physician age/sex brackets was based on data from the 2008 National Survey of Physicians, Dentists, and Pharmacists [19]. We defined practicing physicians as physicians aged 75 years or younger. We calculated the number of practicing physicians per 1,000 population between 2010 and 2035. To estimate practicing physicians' working hours, we referred to data by gender and age provided by the Ministry of Health, Labour, and Welfare of Japan [14]. Male and female physicians worked 85 and 78 hours per week in their late 20 s, and 48 and 40 hours per week in their late 60 s, respectively [14]. In the simulation of the number of physicians, we need to consider the number of newly registered physicians. Let *n_isj_*(*t*) be the number of newly registered physicians of the *i*th prefecture, sex *s,* and *j*th the age group in year *t*. If we know *n_isj_*(*t*), Eq. (1) can be rewritten as
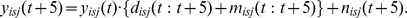



In the simulation for physicians, age groups are defined as follows: The first age group includes physicians aged ≤24 years, and the remaining groups consist of physicians in 5-year intervals (e.g., age 25–29, 35–39, etc.).

We should note that no survey has been done to count the number of newly registered physicians for each tuple of prefecture, sex, and age group. Therefore, we estimated *n_isj_*(*t*) based on the data of the number of physicians in 2008 for each tuple provided by the Ministry of Health, Labour, and Welfare, of Japan [19]. First, we computed the basal level of *n_isj_*(*t*) that is independent of year by
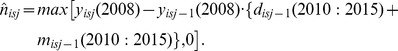



We can consider that 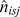 reflects the regular number of new medical school students in Japan around 2001, because a student who entered a medical school in 2002 is expected to have graduated in 2008. However, from 2008 to 2010, the regular number of the first-year medical students increased. To account for this effect, we multiply 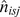 by the constant *c*(*t*) and express the updated equation as
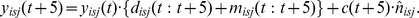



The constant *c*(*t*) was determined by considering the actual regular numbers of first-year medical students in 2008, 2009, and 2010.

To determine working hours of physicians, we referred to data provided by the Ministry of Health, Labour and Welfare, Japan [13]. The data revealed that physicians in their late 20 s worked 85 hours per week. We modeled the working hours pre week, *w_sj_*, for males by
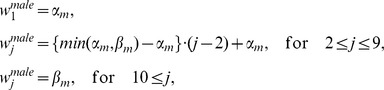
where the age groups corresponding to *j*  = 1 and *j*  = 9 are “≤24 years old” and “≥60and ≤64 years old,” respectively. The working hours per week for females were similarly defined by



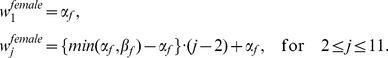



Note that the age group corresponding to *j*  = 11 includes physicians whose age is ≥70 and ≤74 years. In the above model, the working hours per week follows the data of the Ministry of Health, Labour and Welfare, of Japan, by using 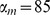, 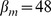, 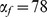, and 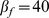. We controlled 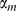 and 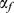 in the simulations limiting working hours per week.

In the simulation of increasing the regular number of new students in medical schools, we defined the increasing rate as *a*; we assumed that the regular number of first-year medical students increases (1+*a*) times that in 2010. We assumed that the policy for increasing regular numbers will be implemented for students who will enter medical schools in April 2013 and we thus revise *a* to zero and 3/5 times in 2015 and 2020, respectively. Note that we consider that the constant, *a*, is a positive value; however, this framework can represent a decrease in the regular number of new students and a more flexible determination of regular numbers by considering the year-dependent constant, *a*(*t*).

We defined two indicators: the number of fatalities per practicing physician and the number of fatalities per practicing physician working hour. A total of 78**·**6% of Japanese people die in hospitals, [20] and the number of fatalities reflects actual healthcare demands. The indicators were estimated for the total population, for subjects aged 74 years or younger, and for subjects aged 75 years or older. The reason for setting a boundary at 75 years was that the average healthy life expectancy (HALE: the average number of years that a person can expect to live in full health by taking into account years lived in less than full health due to disease and/or injury) is 76 years (73 years for males and 78 years for females) in Japan, which is the highest in the world [21]. The percentage of persons who require long-term care increases explosively when they exceed 75 years of age [22].

**Figure 3:**
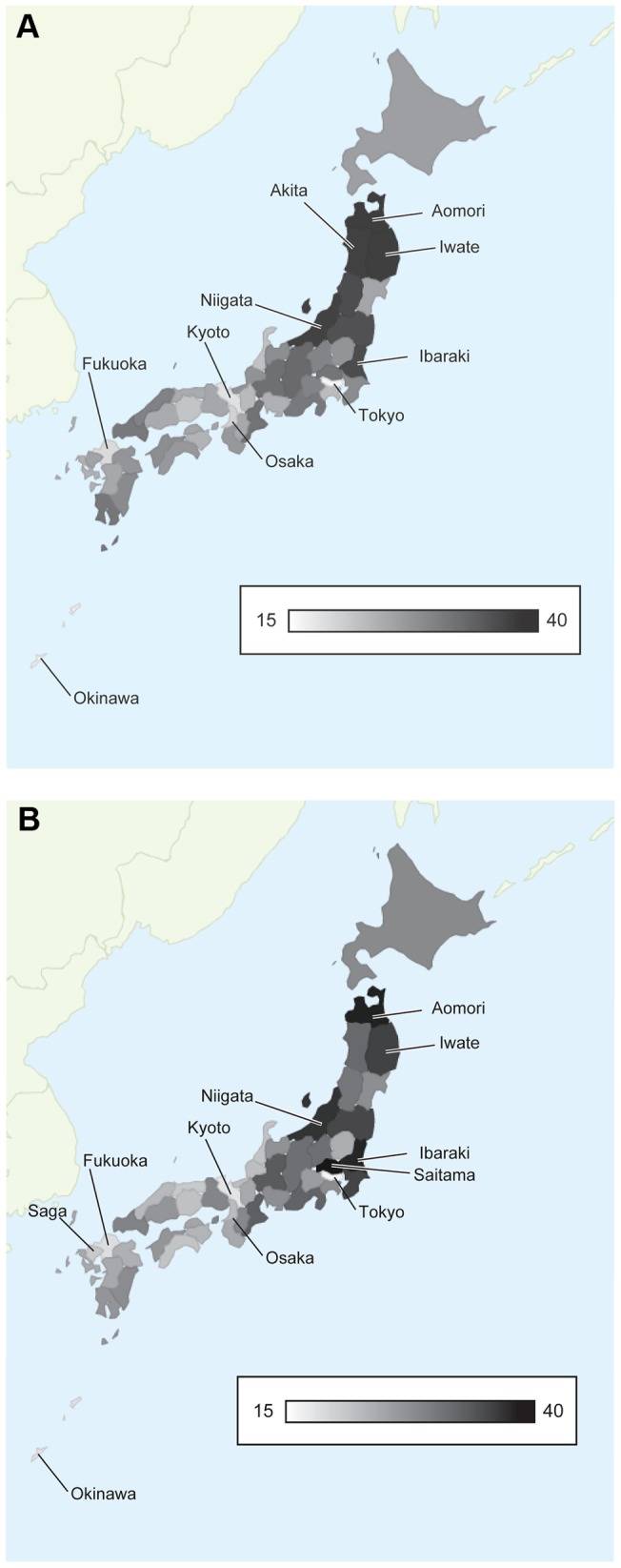
Number of fatalities per practicing physician for the entire population by prefecture. Values for 2010 are calculated and mapped (panel A). The indicator for all of Japan is 24·0. The best five prefectures are Tokyo (15·8), Okinawa (16·5), Kyoto (17·7), Fukuoka (18·2), and Osaka (19·1). The worst five prefectures are Aomori (35·2), Iwate (34·8), Akita (34·3), Niigata (34·2), and Ibaraki (32·7). Values for 2035 are calculated and mapped (panel B). The indicator for all of Japan is 23·1. The best five prefectures are Tokyo (15·7), Kyoto (17·3), Okinawa (17·3), Fukuoka (17·9) and Saga (20·0). The worst five prefectures are Saitama (38·2), Aomori (36·9), Ibraraki (36·1), Niigata (34·1), and Iwate (32·6).

**Figure 4:**
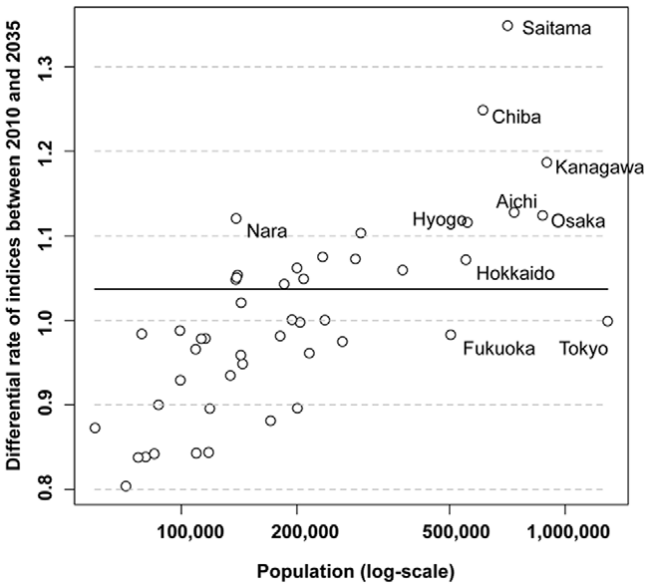
Correlation between prefectural populations and the differential increase in rates in the number of fatalities per physician for the entire population. When fatalities per practicing physician were compared between 2010 and 2035, the differential increase in rates was correlated with prefectural populations.

We calculated serial changes in these indicators between 2010 and 2035, and estimated the required number of new physicians needed to maintain the levels of indicators through the study period. We further calculated serial changes in indicators among the 47 prefectures in Japan. Gray gradation maps of Japan were drawn using Google Visualization API (California, USA).

## Results

### Practicing physicians per 1,000 population

The number of practicing physicians per 1,000 population is predicted to increase from 2·00 in 2010 to 3·14 in 2035. The total number of practicing physicians is predicted to increase from 254,126 in 2010 to 347,103 in 2035, a 37% increase. The number of physicians aged 60 years or older is expected to increase from 55,375 (20% of physicians) in 2010 to 141,711 (36% of physicians) in 2035 (Table 1 and Figure 1).

The number of female physicians is expected to increase from 49,113 (15% of physicians) in 2010 to 99,807 (27% of physicians) in 2035.

### Practicing physicians' working hours per 1,000 population

Practicing physicians' working hours per 1,000 population was predicted to increase from 139·2 hours in 2010 to 209·8 hours in 2035, a 51% increase (Table 1). If working hour regulations were implemented and physicians' working hours are limited to 60 hours and 48 hours per week in 2035, practicing physicians' working hours per 1,000 population were predicted to be 178·2 hours and 147·7 hours respectively, which correspond to a 28% increase and a 6% increase, respectively, compared with 2010.

### Number of fatalities per practicing physician

The total number of fatalities for consecutive five years is predicted to increase from 5,881,151 in 2010 to 8,336,263 in 2035, a 42% increase (Figure 2 and Table 1). The number of fatalities for 75 years and older is predicted to increase from 3,529,540 in 2010 to 6,650,448 in 2035, an 88% increase. The number of fatalities for 74 years or younger is predicted to decrease from 2,351,611 in 2010 to 1,685,815 in 2035, a 28% decrease. The number of fatalities per practicing physician was predicted to increase from 23·1 to 24·0 (4% increase) between 2010 and 2035. The number of fatalities per practicing physician for 75 years or older is predicted to increase from 13·9 in 2010 to 19·2 in 2035, a 38% increase. The number of fatalities per practicing physician for 74 years or younger is predicted to decrease from 9·2 in 2010 to 4·8 in 2035, a 48% decrease.

### Number of fatalities per practicing physician working hour

In 2010 and 2035, the numbers of fatalities per practicing physician working hour (100 hours) were predicted to be 0·128 and 0·138, respectively (Table 1 and S1). This indicator was predicted to increase by 8% in 2035 compared with 2010. However, if working hours were limited to 60 hours per week and 48 hours per week (as per EU limitations), these indicators were predicted to increase by 27% to 0·162 and 53% to 0·196, respectively.

### Geographic distribution of physicians

Disparities by regions are anticipated in 2010 and 2035 (Figure 3 and S1). In 2010, the numbers of fatalities per practicing physician were lowest in Tokyo (15·8) and highest in Aomori (35·2). In 2035, they were lowest in Tokyo (15·7) and highest in Saitama (38·2) (Figure 3). In addition to the rural-urban inequalities, the indicators are predicted to be significantly high in 2035 in overpopulated areas surrounding Tokyo (Figure S2). When fatalities per practicing physician were compared between 2010 and 2035, the differential increase rates were correlated with prefectural populations (Figure 4).

## Discussion

Our simulation showed future surges in healthcare demands due to the rapid aging of the Japanese population. Previous studies [11], [12] have predicted that the number of physicians in Japan per 1,000 population in 2035 will reach the average level in OECD countries for the year 2010, which is consistent with our findings. However, physicians' working hours per population and the fatalities number per physician in 2035 suggests that physicians' supply will not be enhanced as expected by the increase in physician numbers.

One reason for overestimation is the aging of physicians. The aging of practicing physicians is of concern, with 35% of physicians being 60 years or older in 2035 compared with only 20% in 2010. Elderly physicians work much less compared with younger physicians (male and female physicians work 85 and 78 hours per week in their late 20 s, and 48 and 40 hours per week in their late 60 s) [14]. A marked aging of the general practitioners' workforce has been noted in Australia [23] and the Netherlands, [24] but the future impact of physicians' aging has not been evaluated fully. Changes in physicians' age structure should be considered when discussing the future physician workforce.

Another reason for overestimation is the increase in female physicians. The percentage of the female physicians who passed the National Medical Practitioners Qualifying Examination was 33·2% in 2011, and our simulation revealed that the percentage of female physicians is predicted to rise from 15% in 2010 to 27% in 2035. This trend is comparable to other countries [25]–[28]. Our simulation was based on a survey in 2011, and we did not consider future increases in female medical students. We might have underestimated the effect of increases in female physicians. To improve these situations, we should recognize that only 30% of female physicians are reinstated after retirement such as marriage and childbirth [29]. Improvements in working conditions and the work environment will be necessary, especially for female physicians with small children [30], [31].

We first simulated the future physicians' supply based on the physicians' actual working hours. Past projection of physician supply by the Japanese government was only based on the absolute number of physicians and did not focus on physicians' aging or the increase in female physicians. To restore the future “fatalities per physicians' working hours” indicator back to 2010 value, we showed that the number of new physicians needs to be increased by 8%. If working hours are limited to 48 hours per week, the increase rate jumps to 53%. The trends in implementation of work-time restrictions, based on patient safety and physicians' health, should be considered for future physicians' workforce prediction.

Medical innovation might have a huge impact on healthcare demand. The required number of physicians will increase in accordance with medical advancements [32]. Typical examples are gynecologists and pediatricians. In Japan, the number of live births decreased from 2,091,983 in 1973 to 1,071,304 in 2010, while a severe physician shortage is recently documented in pediatrics and obstetrics [14]. These findings suggest that medical renovation affects physicians' demands, and our simulation might underestimate future healthcare demands.

Our simulation predicted future geographic disparities of physicians. Previous reports showed that the geographic disparities between urban and rural areas has widened despite an increase in total physician number [12], [33], [34]. These findings were comparable to our study, and the difference in fatalities numbers between Tokyo (15·7 per physician) and Aomori (35·2 per physician) is predicted to remain in 2035. In addition to the rural-urban inequalities, the physician shortage will be significant in 2035 in overpopulated areas surrounding Tokyo. This is attributable to the mass-migration from rural areas to cities surrounding Tokyo, which occurred during Japan's economic boom after 1960. These migrants will age simultaneously, requiring medical care in future.

How can we tackle the future physician shortage? One solution is to establish new medical schools where physician shortages are severe. The United States, which will have 62,900 fewer doctors than needed in 2015, [35] are now planning to establish six new medical schools [36]. Some Japanese researchers proposed this solution to the Japanese government, but it will be difficult to achieve due to the strong opposition by the Japanese Medical Association [37]. Another solution is to allocate physicians to the shortage areas. Several prefectures are trying to settle residents to the areas with physician shortages by granting a scholarship to medical students, although these indirect measures play limited roles in ameliorating the physician shortage. Under the Japanese social system, neither government nor medical associations have the powers to force physicians to move to different areas.

This study has several limitations. First, we did not account for any possible medical advances. Our simulation might underestimate the necessary number of physicians. Second, the impacts of financial changes were not considered. Healthcare demand and supply are affected by economic status. For example, the Greek parliament approved a package of drastic healthcare cuts after the Greece debt crisis. Constant budget deficits in Japan might cause the same critical situation in the future. Finally, changes in the healthcare system might affect physician supply. The introduction of a new post-graduate clinical training system in 2004 impacted physicians' career paths and geographical distribution, [38] but this effect was not considered in this study.

In conclusion, a physician shortage for the aging society is predicted to be prominent in 2035. The change of age/sex structure of physicians has a negative effect on the workforce. Strategies to increase the number of physicians, improve working conditions, and solve poor distribution of physicians are urgently needed.

## Supporting Information

Figure S1 **Number of physicians per 1,000 population in Japan by prefecture.** Values for 2010 are calculated and mapped (panel A, average: 2·00). The best five prefectures are Tokushima (2·61), Kyoto (2·60), Tokyo (2·56), Fukuoka (2·53), and Kochi (2·53). The worst five prefectures are Saitama (1·31), Ibaraki (1·45), Chiba (1·52), Aomori (1·61), and Niigata (1·63). Values for 2035 are calculated and mapped (panel B, average: 3·14). The best five prefectures are Kochi (4·33), Tokyo (4·30), Kyoto (4·11), Shimane (4·09), and Wakayama (4·02). The worst five prefectures are Saitama (1·97), Ibaraki (2·21), Chiba (2·35), Aomori (2·44), and Niigata (2·45).
(TIF)

Figure S2 **Differential increase in the number of fatalities per physician for the entire population between 2015 and 2035.** The indicator for all of Japan is 104%. The best five prefectures are Shimane (80) and Kochi, Fukui, Saga, Akita (84 each). The best five prefectures are Saitama (135), Chiba (125), Kanagawa (119), Aichi (113) and Osaka (112).
(TIF)

Table S1 Numbers of fatalities per practicing physician working hour in 2010 and 2035.
(XLS)

## References

[1] National Institute of Population and Social Security (2011) Population Projections for Japan:2001–2050. [in Japanese]. Available: http://www.ipss.go.jp/pp-newest/e/ppfj02/top.html. Accessed 2012 Jun17.

[2] Organization for Economic Co-operation and Development (2010) Practising physicians per 1000 population. OECD health data 2010 Available: http://www.oecd.org/document/30/0,3746,en_2649_37407_12968734_1_1_1_37407,00.html. Accessed 2012 Jun 17.

[3] Ministry of Health, Labor and Welfare (2008) Survey of Medical Care Activities on Public Health Insurance. Available: http://www.mhlw.go.jp/english/database/db-hss/dl/shw-03.pdf. Accessed 2012 Jun 17.

[4] Anon (2006) Pediatric care hurt by doctor shortage. The Japan Times. April 12, 2006. Available: http://www.japantimes.co.jp/text/nn20060412f1.html. Accessed 2012 Sep 12.

[5] Anon (2007) Coping with the doctor shortage. The Japan Times. Oct. 1, 2007. Available: http://www.japantimes.co.jp/text/ed20071001a1.html. Accessed 2012 Sep 12.

[6] Anon (2008) Doctor shortage takes a toll in Japan. Agence France Presse. Mar 15, 2008. Available: http://afp.google.com/article/ALeqM5i5XP-O252HC9opxHZ6aKgsXRKjqw. Accessed 2012 Sep 12.

[7] Anon (2008) Doctor shortage gives patients runaround. The Japan Times. April 12, 2008. Available: http://www.japantimes.co.jp/text/nn20080412f2.html. Accessed 2012 Sep 12.

[8] Anon (2009) Shortage of rural doctors worsens. The Japan Times. April 23, 2009. Available: http://www.japantimes.co.jp/text/nn20090423a8.html. Accessed 2012 Sep 12.

[9] Nagamatsu S, Kami M, Nakata Y (2009) Healthcare safety committee in Japan: mandatory accountability reporting system and punishment. Curr Opin Anaesthesiol 22: 199–206. 19390246 10.1097/ACO.0b013e328323f7aa

[10] Matsumoto K, Kitagawa T, Ito S, Seto K, Hasegawa T, et al (2010) Study on supply,demand and distribution of physicians in Japan. Japanese Medical Management Magazine 10: 575–582.

[11] Koike S, Yasunaga H, Matsumoto S, Ide H, Kodama T, et al (2009) A future estimate of physician distribution in hospitals and clinics in Japan. Health policy (Amsterdam, Netherlands) 92: 244–249. 10.1016/j.healthpol.2009.04.005 19481286

[12] Kobayashi Y, Takaki H (1992) Geographic distribution of physicians in Japan. Lancet 340: 1391–1393. 1360099 10.1016/0140-6736(92)92569-2

[13] Takata H, Nagata H, Nogawa H, Tanaka H (2011) The current shortage and future surplus of doctors: a projection of the future growth of the Japanese medical workforce. Hum Resour Health 9: 14. 21619585 10.1186/1478-4491-9-14 PMC3115839

[14] Yazaki Y, Hasegawa T, Yoshimura H, Yamamoto S, Honda M, et al. (2006) A report of the study group on physician supply and demand - survey of working condition of physician. Ministry of Health, Labour and Welfare, Japan. [in Japanese]. Available: http://www.mhlw.go.jp/shingi/2006/03/s0327-2d.html. Accessed 2012 Jun 17.

[15] Hiyama T, Yoshihara M (2008) New occupational threats to Japanese physicians: karoshi (death due to overwork) and karojisatsu (suicide due to overwork). Occupational and environmental medicine 65: 428–9. 10.1136/oem.2007.037473 18487428

[16] Lockley SW, Cronin JW, Evans EE, Cade BE, Lee CJ, et al (2004) Effect of reducing interns' weekly work hours on sleep and attentional failures. N Engl J Med 351: 1829–37. 15509816 10.1056/NEJMoa041404

[17] Moonesinghe SR, Lowery J, Shahi N, Millen A, Beard JD (2011) Impact of reduction in working hours for doctors in training on postgraduate medical education and patients' outcomes: systematic review. BMJ (Clinical research ed) 342: d1580. 10.1136/bmj.d1580 21427046

[18] Volpp KG, Rosen AK, Rosenbaum PR, Romano PS, Even-Shoshan O, et al (2007) Mortality among patients in VA hospitals in the first 2 years following ACGME resident duty hour reform. JAMA 298: 984–92. 17785643 10.1001/jama.298.9.984

[19] Ministry of Health, Labor, and Welfare (2009) Annual Register Number Table, Guidelines on filling and screening FY2008 Survey of Physicians, Dentists and Pharmacists; 2008. [in Japanese]. Available: http://www.mhlw.go.jp/toukei/saikin/hw/ishi/08/index.html. Accessed 2012 Jun 17.

[20] Ministry of Health, Labor and Welfare (2009) The Vital Statistics of Japan, 2008. [in Japanese]. Available: http://www.mhlw.go.jp/toukei/list/81-1a.html. Accessed 2012 Jun 17.

[21] World Health Organization (2010) Healthy life expectancy (HALE) at birth (years), 2007. Available: http://data.un.org/Data.aspx?q=lifeexpectancy&d=WHO&f=MEASURE_CODE%3AWHOSIS_000002. Accessed 2012 Sep 12.

[22] Anon (2001) Condition of Need for Long-Term Care [in Japanese]. White Paper about Aged Society 2011;30. Available: http://www8.cao.go.jp/kourei/whitepaper/w-2011/zenbun/html/s1-2-3-02.html. Accessed 2012 Jun 17.

[23] Charles J, Britt H, Valenti L (2004) The evolution of the general practice workforce in Australia, 1991–2003. The Medical journal of Australia 181: 85–90. 15257645 10.5694/j.1326-5377.2004.tb06181.x

[24] Campbell JL, Mendive J, Timmermans A (2004) Primary care and general practice in Europe: West and South. In: Jones R, Britten N, Culpepper L, Gass D, Grol R, et al., editors. Oxford Textbook of Primary Medical Care. Oxford, UK: Oxford University Press. 65–70.

[25] Gray S, Finlay I, Black C (2005) Women doctors and their careers: what now? The changing UK medical workforce's effect on planning and delivery of services. BMJ (Clinical research ed) 331: 696. 10.1136/bmj.331.7518.696 PMC1226261 16179717

[26] McMurray JE, Cohen M, Angus G, Harding J, Gavel P, et al (2002) Women in medicine: a four-nation comparison. J Am Med Womens Assoc. 57: 185–90. 12405232

[27] Moore W (2002) BMA negotiator calls for more male medical students. BMJ 324: 754. 10.1136/bmj.324.7340.754/b PMC1122700 11923151

[28] Prunier-Poulmaire S, Gadbois C (2001) The French 35-hour workweek: a wide-ranging social change. J Hum Ergol. 30: 41–6. 14564856

[29] Izumi M (2008) Retirement of female physicians. [in Japanese]. Igaku Kyoiku 39: S15.

[30] Koike S, Matsumoto S, Kodama T, Ide H, Yasunaga H, et al (2009) Estimation of physician supply by specialty and the distribution impact of increasing female physicians in Japan. BMC health services research 9: 180. 19811625 10.1186/1472-6963-9-180 PMC2761900

[31] Kaneto C, Toyokawa S, Inoue K, Kobayashi Y (2009) Gender difference in physician workforce participation in Japan. Health policy 89: 115–23. 18586347 10.1016/j.healthpol.2008.05.010

[32] Jackson DB, Sood AK (2011) Personalized cancer medicine-advances and socio-economic challenges. Nature reviews Clinical oncology 8: 735–41. 10.1038/nrclinonc.2011.151 PMC3435438 21989071

[33] Matsumoto M, Inoue K, Bowman R, Noguchi S, Kajii E (2010) Physician scarcity is a predictor of further scarcity in US, and a predictor of concentration in Japan. Health policy 95: 129–36. 20004995 10.1016/j.healthpol.2009.11.012

[34] Horev T, Pesis-Katz I, Mukamel DB (2004) Trends in geographic disparities in allocation of health care resources in the US. Health policy 68: 223–32. 15063021 10.1016/j.healthpol.2003.09.011

[35] The Association of American Medical Colleges (2012) 2011 State Physician Workforce Data Book. Available: https://www.aamc.org/download/263512/data/statedata2011.pdf. Accessed 2012 Sep 12.

[36] Whitcomb ME (2010) New medical schools in the United States. The New England journal of medicine 362: 1255–8. 20375402 10.1056/NEJMp0912179

[37] Anon (2011) Existential fear stalks M.D.s. The Japan Times. Nov 28, 2011. Available: http://www.japantimes.co.jp/text/eo20111128a1.html. Accessed 2012 Jun 17.

[38] Working Group on the new post-graduate clinical training system, Ministry of Health, Labor and Welfare (2012) The regional impact of introduction of the new post-graduate clinical training system. 2012. Available: http://www.mhlw.go.jp/stf/shingi/2r98520000020kbe-att/2r98520000020kec.pdf. Accessed 2012 Jun 17.

