# Supplementary figures and images for: Forecasting Japan's Physician Shortage in 2035 as the First Full-Fledged Aged Society
Source: PLoS One. 2012 Nov 30;7(11):e50410. doi: 10.1371/journal.pone.0050410 (PMC3511523; doi:10.1371/journal.pone.0050410)

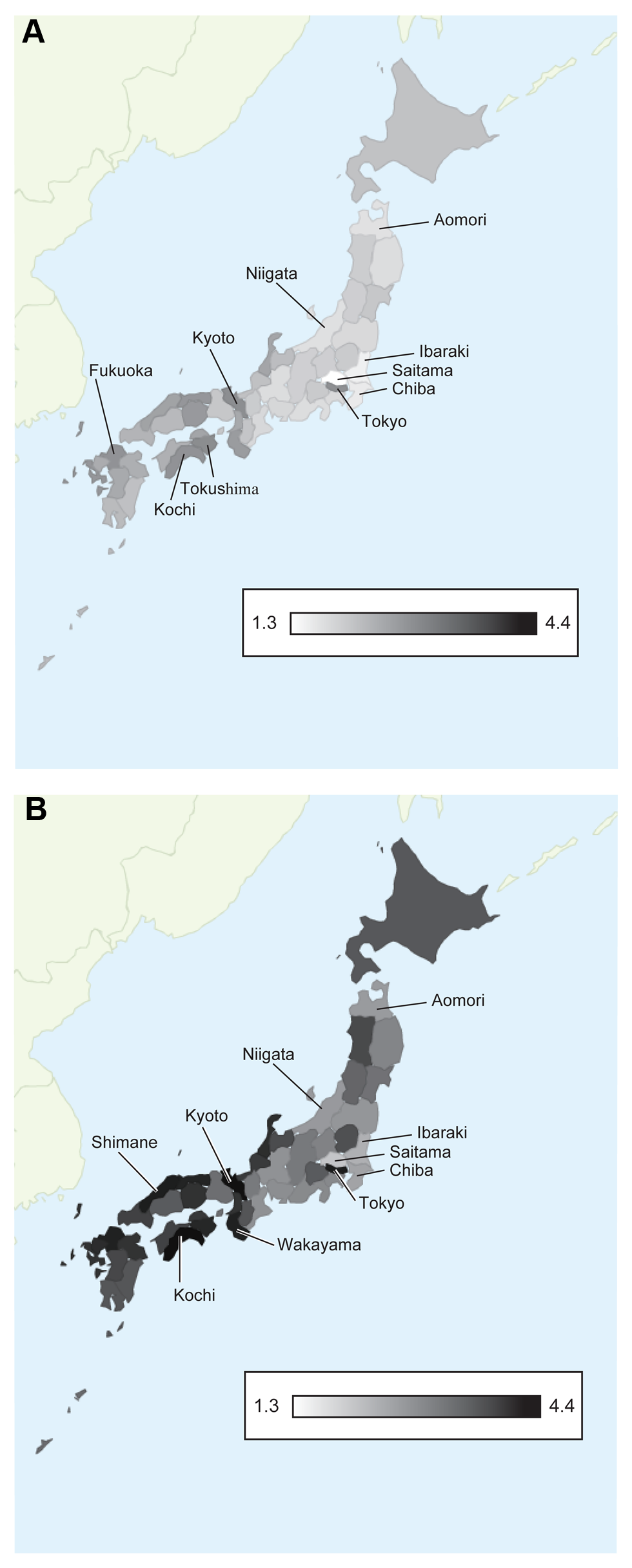

Supplement: Figure S1 — Number of physicians per 1,000 population in Japan by prefecture. Values for 2010 are calculated and mapped (panel A, average: 2·00). The best five prefectures are Tokushima (2·61), Kyoto (2·60), Tokyo (2·56), Fukuoka (2·53), and Kochi (2·53). The worst five prefectures are Saitama (1·31), Ibaraki (1·45), Chiba (1·52), Aomori (1·61), and Niigata (1·63). Values for 2035 are calculated and mapped (panel B, average: 3·14). The best five prefectures are Kochi (4·33), Tokyo (4·30), Kyoto (4·11), Shimane (4·09), and Wakayama (4·02). The worst five prefectures are Saitama (1·97), Ibaraki (2·21), Chiba (2·35), Aomori (2·44), and Niigata (2·45). (TIF) [file pone.0050410.s001.tif]

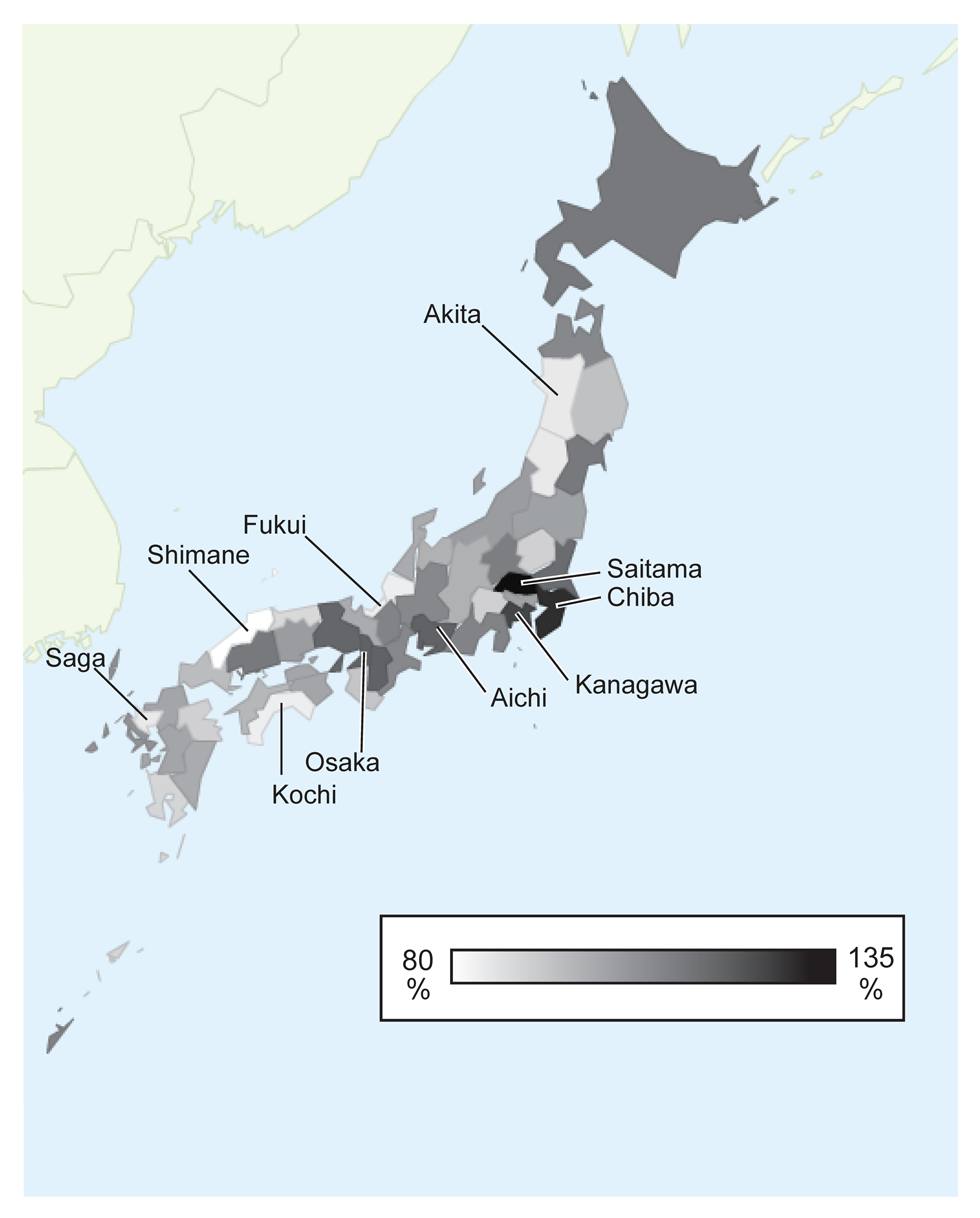

Supplement: Figure S2 — Differential increase in the number of fatalities per physician for the entire population between 2015 and 2035. The indicator for all of Japan is 104%. The best five prefectures are Shimane (80) and Kochi, Fukui, Saga, Akita (84 each). The best five prefectures are Saitama (135), Chiba (125), Kanagawa (119), Aichi (113) and Osaka (112). (TIF) [file pone.0050410.s002.tif]
